# *Gyrodactylus magadiensis* n. sp. (Monogenea, Gyrodactylidae) parasitising the gills of *Alcolapia grahami* (Perciformes, Cichlidae), a fish inhabiting the extreme environment of Lake Magadi, Kenya

**DOI:** 10.1051/parasite/2019077

**Published:** 2019-12-20

**Authors:** Quinton Marco Dos Santos, John Ndegwa Maina, Annemariè Avenant-Oldewage

**Affiliations:** 1 Department of Zoology, University of Johannesburg PO Box 524, Auckland Park 2006 Johannesburg South Africa

**Keywords:** Lake Magadi, *Alcolapia grahami*, *Gyrodactylus*, Kenya, Soda Lake

## Abstract

A new species of *Gyrodactylus* von Nordmann, 1832 is described from the gills of *Alcolapia grahami*, a tilapian fish endemic to Lake Magadi. This alkaline soda lake in the Rift Valley in Kenya is an extreme environment with pH as high as 11, temperatures up to 42 °C, and diurnal fluctuation between hyperoxia and virtual anoxia. Nevertheless, gyrodactylid monogeneans able to survive these hostile conditions were detected from the gills the Magadi tilapia. The worms were studied using light microscopy, isolated sclerites observed using scanning electron microscopy, and molecular techniques used to genetically characterize the specimens. The gyrodactylid was described as *Gyrodactylus magadiensis* n. sp. and could be distinguished from other *Gyrodactylus* species infecting African cichlid fish based on the comparatively long and narrow hamuli, a ventral bar with small rounded anterolateral processes and a tongue-shaped posterior membrane, and marginal hooks with slender sickles which are angled forward, a trapezoid to square toe, rounded heel, a long bridge prior to reaching marginal sickle shaft, and a long lateral edge of the toe. The species is also distinct from all other *Gyrodactylus* taxa based on the ITS region of rDNA (ITS1–5.8s–ITS2), strongly supporting the designation of a new species. These findings represent the second record of *Gyrodactylus* from Kenya, with the description of *G. magadiensis* bringing the total number of *Gyrodactylus* species described from African cichlids to 18.

## Abstract (fr)

Une nouvelle espèce de *Gyrodactylus* von Nordmann, 1832 est décrite à partir des branchies d’*Alcolapia grahami*, un tilapia endémique du lac Magadi. Ce lac de soude alcaline dans la vallée du Rift au Kenya est un environnement extrême avec un pH allant jusqu’à 11, des températures allant jusqu’à 42 °C et des fluctuations diurnes entre l’hyperoxie et l’anoxie virtuelle. Néanmoins, des Monogènes Gyrodactylidae capables de survivre dans ces conditions hostiles ont été détectés sur les branchies du tilapia de Magadi. Les vers ont été étudiés par microscopie optique, les sclérites isolés ont été observés au microscope électronique à balayage et des techniques moléculaires ont été utilisées pour caractériser génétiquement les spécimens. Le gyrodactylidé est décrit comme étant *Gyrodactylus magadiensis* n. sp. et se distingue des autres espèces de *Gyrodactylus* infectant les cichlidés d’Afrique grâce à ses hamuli relativement longs et étroits, à une barre ventrale avec de petits processus antérolatéraux arrondis et à une membrane postérieure en forme de langue, ainsi qu’à des crochets marginaux à faucilles minces inclinées vers l’avant, un trapèze à bout carré, un talon arrondi, un long pont avant d’atteindre la faucille marginale et un long bord latéral de l’extrémité. L’espèce est également distincte de tous les autres taxons de *Gyrodactylus* sur la base sur la région ITS de l’ADNr (ITS1–5.8s–ITS2), ce qui soutient fortement la désignation d’une nouvelle espèce. Ces découvertes représentent la seconde mention d’un *Gyrodactylus* au Kenya, et la description de *G. magadiensis* amène à 18 le nombre total d’espèces de *Gyrodactylus* décrites parmi les cichlidés d’Afrique.

## Introduction

Fishes of the genus *Alcolapia* Thys van den Audenaerde, 1969 are African cichlids that occur in two of the most severe environments in the eastern African Rift Valley, namely the Soda Lakes Magadi and Natron, located in Kenya and Tanzania, respectively [53, 67]. These two lakes were once a continuous water body, separated ~10,000 years ago [7, 14, 22, 52]. Fish subsist in scattered lagoons and sites at the periphery of Lake Magadi where particularly extreme conditions occur, which include pH up to 11, titration alkalinity >300 mM, osmolarity 525 mOsm, temperatures as high as 42 °C, and O_2_ levels fluctuating diurnally between hyperoxia during the day and virtual anoxia at night [5, 28, 39, 41, 46, 53, 56, 61, 66, 68, 69]. Being a closed lake, the alkaline lagoons are recharged by hot geothermal springs where temperatures can reach as high as 86 °C. Despite these hostile conditions, a single species of cichlid fish, *Alcolapia grahami* (Boulenger, 1912), or the Magadi tilapia, inhabits the hot and highly alkaline waters of this lake [41, 62]. The Magadi tilapia has developed exceptional morphological and physiological adaptations, especially to cope with the high pH, extreme alkalinity, and the shifting oxygen availability. These include: (a) excretion of nitrogenous waste in the form of urea instead of ammonia through the ornithine-urea cycle [69], (b) an atypically thin blood–water barrier in the gills [38, 40], and (c) the use of the swim bladder as a primitive air-breathing organ [40].

Gills of African cichlids are often infected by monogenean parasites of the genera *Gyrodactylus* von Nordmann, 1832 and *Cichlidogyrus* Paperna, 1960. Using the conventional estimate of, on average, 1 monogenean species per fish host, the projected international diversity of the genus *Gyrodactylus* was proposed to be 20,000 species [4, 24]. Nevertheless, the number of *Gyrodactylus* spp. from African freshwater fish amounts to 35 [57], of which only 17 are from cichlids (Table 1). Thus, the species currently known denote only a fragment of the number of anticipated *Gyrodactylus* spp. in Africa, especially from cichlids. In contrast, even though *Gyrodactylus* is predicted to be more specious, substantially more species of *Cichlidogyrus* have been described from African cichlids (122 according to Řehulková et al. [57]). Within *Gyrodactylus*, several morphological criteria are used to discriminate between species. These include metrics of haptoral sclerites, the form and shape of marginal hooks, the presence of additional haptoral elements (e.g. ventral bar processes), the number and arrangement of the MCO spines, and the morphology, and shape of the ventral bar membrane (e.g. [20, 26, 64]). The use of molecular approaches to support taxonomic studies of these parasites have also been implemented in the past few decades, but genetic data for several species are not yet available. Regarding the 17 species of *Gyrodactylus* from cichlids, for example, sequence data for only 13 are currently available. Only a single *Gyrodactylus* species has been described from Kenya, *Gyrodactylus malalai* Přikrylová, Blažek and Gelnar, 2012 from *Oreochromis niloticus niloticus* (L.) and *Tilapia zillii* (Gervais) in Lake Turkana.

**Table 1:** Collection details, including author, host, and distribution, for all species of *Gyrodactylus* von Nordmann, 1832 described and recorded from African cichlids. Type hosts in bold.

Species	Authors	Host	Country	Reference
G. aegypticus*	El-Naggar and El-Tantawy, 2003	***Coptodon zillii* (Gervais)**	Egypt	[12]
*G. chitandiri*	Zahradníčková, Barson, Luus-Powell and Přikrylová, 2016	***Coptodon rendalli* (Boulenger)**	Chirundu, Zambezi River, and Lake Kariba, Zimbabwe	[70]
		*Pseudocrenilabrus philander* (Weber)		
*G. cichlidarum*	Paperna, 1968	***Sarotherodon galilaeus* (L.)**	Accra Plain and Akuse Lagoon, Ghana	[49]
		*Hemichromis fasciatus* Peters	Accra Plain and Akuse Lagoon, Ghana	[49]
		*Hemichromis bimaculatus* Gill		
		*Coptodon zillii* (Gervais)		
		*Sarotherodon galilaeus* (L.)	Accra Plain and Akuse Lagoon, Ghana	[51]
		*Sarotherodon melanotheron* Rüppell		
		*Coptodon guineensis* (Günther)		
		*Coptodon zillii* (Gervais)		
		*Hemichromis fasciatus* Peters	Mare Simenti, Niokolo Koba National Park, Senegal	[54]
		*Oreochromis niloticus* (L.)	Cultured stock, University of Stirling, UK	[15]
		*Astronotus ocellatus* (Agassiz)	Various pet stores, Tehran, Iran	[45]
		*Poecilia mexicana* Steindachner	Puebla and Michoacán, Mexico	[19]
		*Poeciliopsis gracilis* (Heckel)		
		*Pseudoxiphophorus bimaculatus* (Heckel)		
		*Oreochromis niloticus* (L.)	University of the Philippines, Visayas, Iloilo Province, Philippines	[9]
		*Oreochromis niloticus* (L.)	Vietnam Mekong River Delta	[2]
*G. ergensi*	Přikrylová, Matějusová, Musilová and Gelnar, 2009	***Sarotherodon galilaeus* (L.)**	Mare Simenti, Niokolo Koba National Park, Senegal	[54]
		*Oreochromis niloticus* (L.)		
*G. haplochromi*	Paperna, 1973	***Haplochromis angustifrons* Boulenger**	Lake George, Uganda	[50]
*G. hildae*	García-Vásquez, Hansen, Christison, Bron, and Shinn, 2011	***Oreochromis niloticus* (L.)**	Tributary of the Baro River, Gambela, Ethiopia	[16]
*G. malalai*	Přikrylová, Blažek, and Gelnar, 2012	***Oreochromis niloticus* (L.)**	Lake Turkana, Kenya	[55]
		*Coptodon zillii* (Gervais)		
		*Oreochromis niloticus* (L.)	Blue Nile, Sudan	[55]
*G. nyanzae*	Paperna, 1973	***Oreochromis variabilis* (Boulenger)**	Lake Victoria, Uganda	[50]
		*Oreochromis niloticus* (L.) X	Kipopo station, Haut-Katanga province, DRC	[65]
		*Oreochromis mweruensis* Trewavas		
		*Coptodon rendalli* (Boulenger)		
		*Oreochromis niloticus* (L.)	Chirundu, Zambezi River, Zimbabwe	[70]
		*Oreochromis mweruensis* Trewavas	Kipopo and Luapula River, Bangweulu-Mweru, DRC	[29]
		*Coptodon rendalli* (Boulenger)		
*G. occupatus*	Zahradníčková, Barson, Luus-Powell and Přikrylová, 2016	***Oreochromis niloticus* (L.)**	Lake Chivero, Zimbabwe	[70]
		*Pharyngochromis acuticeps* (Steindachner)		
		*Pseudocrenilabrus philander* (Weber)		
*G. parisellei*	Zahradníčková, Barson, Luus-Powell and Přikrylová, 2016	***Oreochromis niloticus* (L.)**	Lake Chivero and Lake Kariba, Zimbabwe	[70]
		*Pseudocrenilabrus philander* (Weber)		
*G. shariffi*	Cone, Arthur and Bondad-Reantaso, 1995	***Oreochromis niloticus* (L.)**	University of the Philippines, Visayas, nIloilo Province, Philippines	[9]
*G. sturmbaueri*	Vanhove, Snoeks, Volckaert and Huyse, 2011	***Simochromis diagramma* (Günther)**	Kalambo Lodge, Lake Tanganyika, Zambia	[64]
		*Pseudocrenilabrus philander* (Weber)	Chirundu, Zambezi River, Zimbabwe	[70]
*G. thlapi*	Christison, Shinn and van As, 2005	***Pseudocrenilabrus philander* (Weber)**	Shakawe, Sepopa and Seronga, Okavango Delta, Botswana	[8]
		*Pseudocrenilabrus philander* (Weber)	Baberspan Wetland, South Africa	[63]
*G. thysi*	Vanhove, Snoeks, Volckaert and Huyse, 2011	***Simochromis diagramma* (Günther)**	Kalambo Lodge, Lake Tanganyika, Zambia	[64]
*G. ulinganisus*	García-Vásquez, Hansen, Christison, Bron and Shinn, 2011	***Oreochromis mossambicus* (Peters)**	Welgevallen, Stellenbosch, South Africa	[16]
*G. yacatli*	García-Vásquez, Hansen, Christison, Bron and Shinn, 2011	***Oreochromis niloticus* (L.)**	Gania de Pucté, Municipal de Chablé, Tabasco, Mexico	[16]
			Merida, Mexico	
			Culiacan, Mexico	
		*Oreochromis niloticus* (L.)	Chirundu, Zambezi River, Zimbabwe	[70]
		*Pseudocrenilabrus philander* (Weber)		
*G. zimbae*	Vanhove, Snoeks, Volckaert and Huyse, 2011	***Simochromis diagramma* (Günther)**	Kalambo Lodge, Lake Tanganyika, Zambia	[64]
		*Ctenochromis horei* (Günther)		

* *Nomen nudum*.

Monogenean parasites have previously been recorded from systems with extreme physicochemical environments, such as *Gyrodactylus salinae* Paladini, Huyse and Shinn 2011 from the hypersaline Cervia Saline in Italy [47], and as such may occur alongside the highly adapted Magadi tilapia. Specimens of *A. grahami* collected at the Fish Springs Lagoon of Lake Magadi provided a unique opportunity to study this species for possible parasite infections. In doing so, an unidentified species of *Gyrodactylus*, adapted to, and thriving, in these extreme conditions, was detected. Thus, this paper represents the morphological and molecular description of a new species of *Gyrodactylus*, and the first record of a gyrodactylid parasite from the gills of the Lake Magadi tilapia, *A. grahami* from Kenya.

## Materials and methods

### Collection

**Figure 1:**
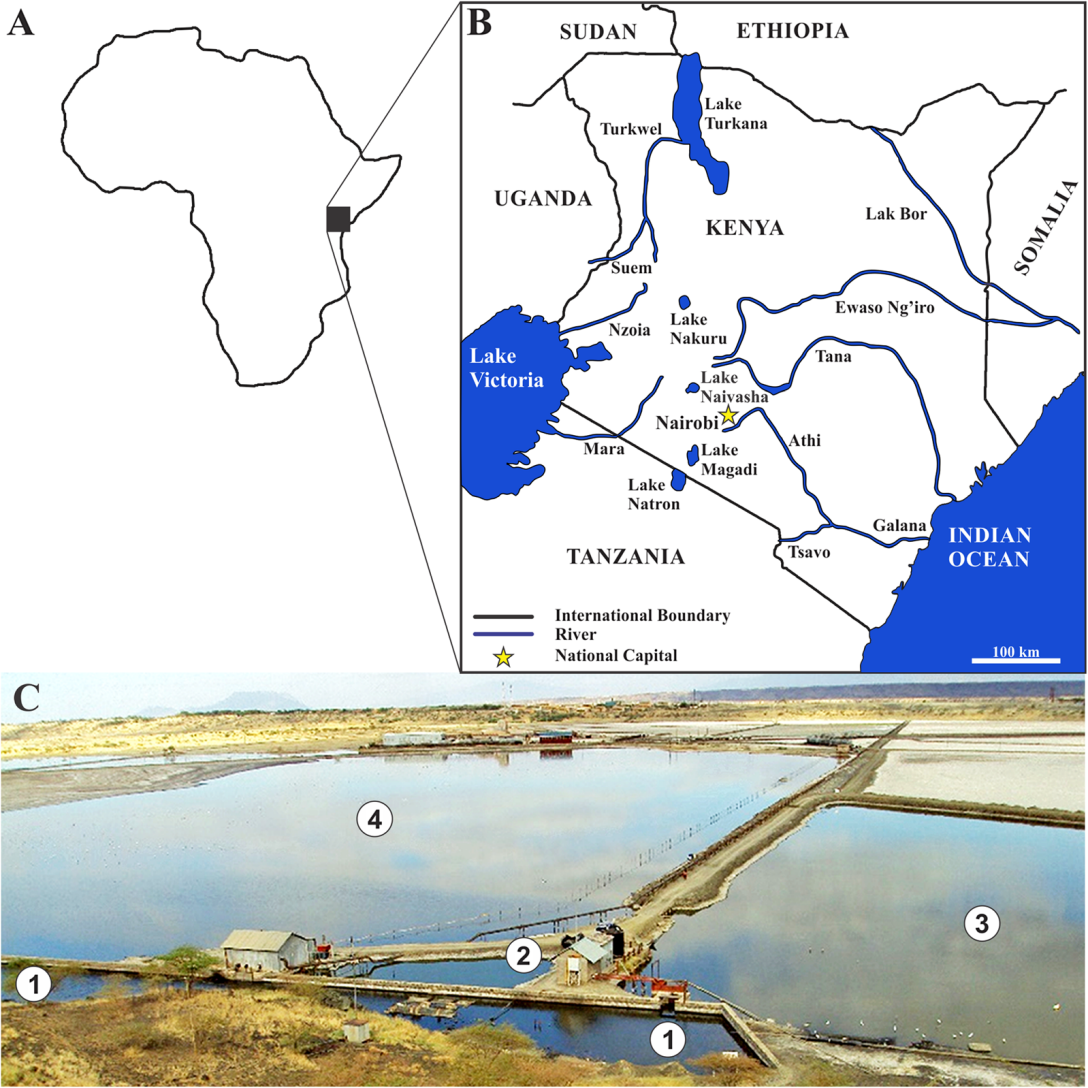
Collection sites from which *Alcolapia grahami* were collected. (A) Silhouette of Africa showing area of study; (B) map of study area indicating countries, water bodies, and relation of Lake Magadi to Nairobi; (C) fish spring lagoon of Lake Magadi from which the fish specimens were collected.

Fish were collected (permit number NCST/RRI/12/1/MAS/99) from the Fish Spring Lagoon of Lake Magadi (Fig. 1B and C) in July 2013 and June 2018. Fish were specifically collected from the most peripheral pool (1 in Fig. 1C). Fish were euthanised on site by severing the spinal cord and the whole fish preserved in either formalin (July 2013) or absolute ethanol (June 2018). At the University of Johannesburg, gills were removed from the preserved fish and studied for the presence of parasites, with attached worms removed from the gills using fine dissecting needles and detached worms picked carefully from the fixative.

### Morphometric analyses

Formalin fixed worms were washed in water, dehydrated in a series of ethanol (30%, 50%, and 70% ethanol), and subsequently mounted and cleared in glycerine ammonium picrate (GAP) [42] for examination of the haptoral sclerites and male copulatory organ (MCO). Some specimens were also stained with Horen’s trichrome [43] and cleared and mounted in lactophenol. Light microscopy, using both phase and differential interference contrast approaches, were used to study the shape and dimensions of sclerotized structures using a Zeiss Axioplan 2 imaging light microscope with Axiovision 4.7.2 software (Carl Zeiss, Jena, Switzerland). Micrographs were used to draw taxonomically important structures. The measurements of hamuli and other haptoral sclerites (28 point-to-point measurements) were taken according to Shinn et al. [60] and García-Vásquez et al. [17], and the measurements of the parasite’s whole body were taken according to Christison et al. [8]. All measurements are in micrometres unless stated otherwise, and presented as a mean and range in parentheses. Measurements of the haptoral sclerites are presented in full alongside the only other *Gyrodactylus* species described from Kenya (*G. malalai*) in Table 2. After analyses, worms were removed from the slides, dehydrated through a graded ethanol series, and mounted in Canada balsam for permanent storage [13].

**Table 2:** Morphological measurements of *Gyrodactylus magadiensis* n. sp. from the Magadi tilapia, *Alcolapia grahami* collected from Lake Magadi, Kenya. Data are presented alongside data for the only other previously described *Gyrodactylus* species from Kenya. Data compiled from previous study directly transposed as noted by author.

Measurement	*G. magadiensis* n. sp.	*G. malalai*
	*n* = 24	*n* = 18
	Present study	Přikrylová et al. [55]
Total body length	267 ± 53 (202–387)	792 ± 74.8 (666–876)
Total body width	65 ± 17.6 (41.4–100)	118 ± 13.7 (98–136)
Pharynx length	21.1 ± 4.6 (13.8–29.7)	42.5 ± 6.2 (34–49.5)
Pharynx width	19.7 ± 4 (13.6–28.2)	37.2 ± 6.4 (26.5–44)
Posterior pharynx length	15.6 ± 2 (13.5–18)	
Posterior pharynx width	13.2 ± 2.2 (11–15)	
MCO length	11.3 ± 1.9 (9.6–14)	
MCO width	8.5 ± 1.3 (6.9–10.2)	
MCO spines	1L, 6S	1L, 4–5S
Hamulus		
Total length	62.8 ± 5.6 (53.6–73.7)	109 ± 3.9 (102–116.5)
Aperture	31 ± 2.2 (27.3–35.3)	
Point shaft width	6.9 ± 0.9 (5.3–8.7)	
Point length	24.2 ± 2.4 (19.9–29.5)	40.2 ± 2.9 (36–49)
Distal shaft width	3.5 ± 0.5 (2.6–4.6)	
Shaft length	38.7 ± 2.2 (34.9–43.6)	74.5 ± 2.8 (68.5–78)
Inner curve length	24.9 ± 2.3 (21.4–30.2)	
Aperture angle	60 ± 7.4 (50.1–78.1)	
Point curve angle	23.7 ± 5.4 (11.1–36.4)	
Inner aperture angle	66.8 ± 8.5 (55.9–85.5)	
Root length	22.3 ± 3.4 (16.9–29.9)	45 ± 5.5 (32.5–54)
Ventral bar		
Length	27.5 ± 2.7 (22.6–33.3)	
Width	20.8 ± 3.5 (14.6–26.4)	16 ± 1.5 (23.5–28.5)
Process to mid–length	3.5 ± 0.9 (2.1–5.1)	
Mid–length	5.2 ± 1 (3.5–7)	8.3 ± 0.6 (7.5–9.5)
Process length	3 ± 0.5 (2–4)	
Membrane length	18.7 ± 1.6 (15.9–22.8)	14.5 ± 1.4 (12.5–16.5)
Dorsal bar		
Length	2.6 ± 0.5 (2.1–3.6)	2.3 ± 0.2 (2–2.5)
Width	13 ± 1.9 (9.8–17.8)	23.5 ± 1.1 (22.5–25)
Marginal hook		
Total length	27.7 ± 2.5 (23.4–31)	32 ± 0.6 (31.5–33)
Shaft length	23.1 ± 2.3 (18.9–26)	23.5 ± 0.7 (23–24.5)
Sickle length	5.4 ± 0.4 (4.7–6.1)	8.5 ± 0.3 (8–9)
Sickle point width	4 ± 0.5 (3–4.7)	6 ± 0.3 (5.5–6.5)
Toe length	1.8 ± 0.2 (1.5–2.2)	
Sickle distal width	3.4 ± 0.3 (2.9–4)	7.3 ± 0.5 (6.5–8)
Aperture	4.4 ± 0.5 (3.6–5.1)	8 ± 0.5 (7.5–9)
Instep/arch height	0.8 ± 0.1 (0.6–1)	
Filament loop	8 ± 0.8 (6.5–9.2)	

Due to the fixation of samples in formalin and high concentration ethanol, SEM of whole worms did not produce viable results. To study the sclerites at higher magnification, worms were digested on a concavity slide using the digestion buffer from a DNA extraction kit, digested tissue removed, rinsed, and prepared for SEM [10, 11, 37, 44, 60]. Slides were gold sputter coated using an Emscope SC500 Sputter Coater (Quorum Technologies, Newhaven, UK) and studied with using a Vega 3 LMH scanning electron microscope (Tescan, Brno, Czech Republic) at 3.4 kV.

### Molecular characterisation

For genomic identification, 10 ethanol fixed specimens were rehydrated, digested, and genomic DNA extracted using a NucleoSpin^®^ Tissue kit (Macherey-Nagel, Düren, Germany), following the manufacturer’s protocols. The region of rDNA spanning the 3′ end of 18S, ITS1, 5.8S, ITS2, to the 5′ end of 28S was targeted using primers BD1 (5′–GTC GTA ACA AGG TTT CCG TA–3′) and BD2 (5′–TAT GCT TAA (G/A) TT CAG CGG GT–3′) [36]. PCR was performed under the following conditions: initial denaturation at 94 °C for 5 min, 30 cycles of 94 °C for 30 s, 56 °C for 30 s, and 72 °C for 1 min, followed by a final extension at 72 °C for 5 min as per Luo et al. [35]. Successful amplification was verified using 1% agarose gel impregnated with GelRed (Biotuim, Hayward, USA), and amplicons where sequenced using BigDye v3.1 chemistry (Applied Biosystems, Foster City, USA), following Avenant-Oldewage et al. [3]. Sequencing was performed on an ABI3730 automated sequencer (Applied Biosystems, Foster City, USA). Electropherograms were inspected and edited manually (if required) using Geneious R6 [32] and deposited in GenBank. Obtained sequences were aligned to sequence data for the 10 most closely related *Gyrodactylus* species as determined using BLASTn [1]. All sequences used (Table 3) were aligned using MAFFT [30, 31] via the EMBL-EBI portal. Aligned sequence data were analysed using MEGA 7 [34]. Genetic divergence among species of *Gyrodactylus* was estimated using uncorrected *p-*distances. Phylogenies were constructed with maximum likelihood (ML) methods, using the Tamura three-parameter model (5 categories [+G, parameter = 0. 3415]) (determined by the Model Selection tool in MEGA 7) as implemented in MEGA 7 [34]. Analyses were subject to 1000 bootstrap replicate variance estimation. Bayesian inference (BI) analyses were performed with BEAST v2.5.0 [6], using 10 million Markov chain Monte Carlo (MCMC) generations. BI data was prepared using BEAUTi 2, BEAST results assessed using TRACER v1.7.1, and trees processed using TreeAnnotator v2.5.0 and edited using FigTree v1.4.3.

**Table 3:** List of *Gyrodactylus* species included in the phylogenetic analyses with their hosts, collection site, GenBank accession number, and reference.

Species	Host	Locality	GenBank	Reference
*Gyrodactylus branchicus*	*Gasterosteus aculeatus*	Bothnian Bay, Oulu, Finland	AY061977	[71]
*Gyrodactylus branchicus*	*Gasterosteus aculeatus*	Schelde River, Doel, Belgium	AF156669	[72]
*Gyrodactylus rarus*	*Pungitius pungitius*	Bothnian Bay, Oulu, Finland	AY061976	[71]
*Gyrodactylus rarus*	*Pungitius pungitius*	Wilkojadka River, Baltic Sea basin, Poland	FJ435193	[58]
*Gyrodactylus rarus*	*Spinachia spinachia*	Trondheim Biological, Station, Norway	AY338445	[27]
*Gyrodactylus perlucidus*	*Zoarces viviparus*	Manndalselva River, Barents Sea basin, Norway	FJ435202	[58]
*Gyrodactylus medaka*	*Oryzias latipes*	Midori River, Kumamoto, Japan	LC368478	[47]
*Gyrodactylus medaka*	*Oryzias latipes*	Nuta River, Hiroshima, Japan	LC368475	[47]
*Gyrodactylus medaka*	*Oryzias latipes*	AORI Laboratories, University of Tokyo, Tokyo	LC368479	[47]
*Gyrodactylus medaka*	*Oryzias latipes*	Sonose River, Tokushima, Japan	LC368477	[47]
*Gyrodactylus alexgusevi*	*Lota lota*	Oulujoki, Oulu, Finland	AY061979	[71]
*Gyrodactylus groenlandicus*	*Myoxocephalus scorpius*	Little Tancook Island, Nova Scotia, Canada	KJ095104	[33]
*Gyrodactylus alexanderi*	*Gasterosteus aculeatus*	Lake Skagvatn, South Trondelag County, Norway	JN695633	[23]
*Gyrodactylus alexanderi*	*Gasterosteus aculeatus*	Nanaimo, British Columbia, Canada	JF836144	[21]
*Gyrodactylus alexanderi*	*Gasterosteus aculeatus*	Horne Lake, Strait of Georgia, Pacific Ocean basin, Canada	FJ435201	[58]
*Gyrodactylus wilkesi*	*Trematomus bernacchi*	Prince Gustav Channel, Weddell Sea, Antarctica	LT719091	[25]
*Gyrodactylus lamothei*	*Girardinichthys multiradiatus*	San Nicolas Peralta, Lerma river basin, Mexico	KX555668	[59]
*Gyrodactylus lamothei*	*Girardinichthys multiradiatus*	San Nicolas Peralta, Lerma river basin, Mexico	KX555667	[59]
*Gyrodactylus lamothei*	*Girardinichthys multiradiatus*	San Nicolas Peralta, Lerma river basin, Mexico	KX555666	[59]
*Gyrodactylus katamba*	*Goodea atripinnis*	Santiago Mezquititlan, Queretaro, Mexico	KR815854	[18]

## Results

### *Gyrodactylus magadiensis* n. sp.

urn:lsid:zoobank.org:act:89E29993-EC14-4AA3-AAE2-C569D91B5FBD

Type host: *Alcolapia grahami* (Boulenger, 1912) (Perciformes, Cichlidae)

Type locality: Lake Magadi, Eastern Rift Valley, Kenya (1°53′28.4″ S, 36°18′09.6″ E)

Infection site: Gills

Type material: Holotype: Mounted in Canada Balsam and deposited in the Iziko South African Museum, Cape Town, South Africa (accession no. SAM – A091374). Paratypes: four specimens deposited in the Iziko South African Museum, Cape Town, South Africa (accession no. SAMC – A091375 to SAMC – A091378); four specimens deposited in the Natural History Museum, London, UK (accession nos. NHMUK 2019.12.6.1 to NHMUK 2019.12.6.4); and four specimens deposited in the Royal Museum for Central Africa in Tervuren, Belgium (accession nos. M.T.39080 to M.T.390803).

ITS rDNA sequences: Representative sequence submitted to GenBank (accession no. MN738699).

Etymology: The species is named after Lake Magadi from which the specimens were collected.

### Morphological description (Figs. 2 and 3, Table 3)

**Figure 2:**
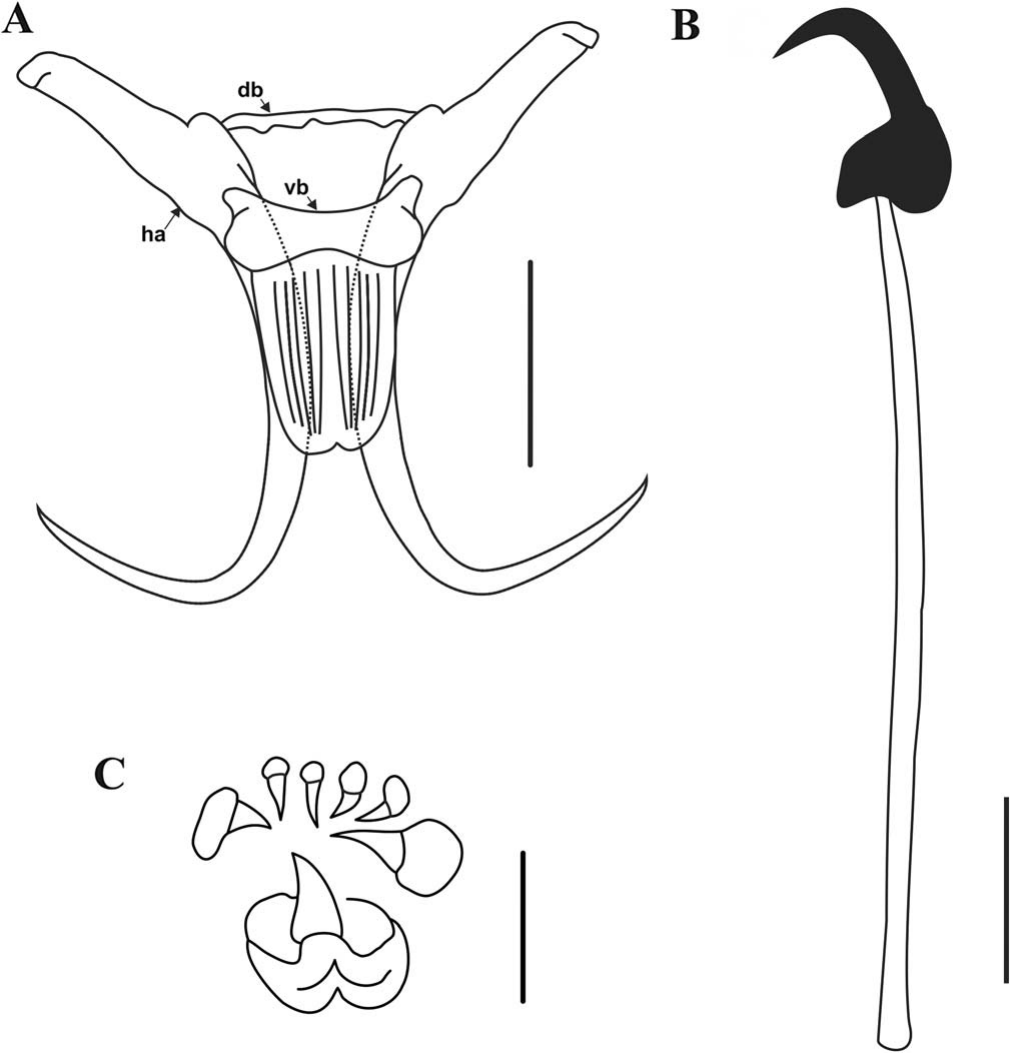
Line drawings of the haptoral sclerites and MCO of *Gyrodactylus magadiensis* n. sp. from *Alcolapia grahami* in Lake Magadi, Kenya. (A) Haptoral sclerites with hamulus (ha), dorsal bar (db), and ventral bar (vb); (B) marginal hook; (C) male copulatory organ (MCO). Scale bars – (A) 20 μm; (B and C) 5 μm.

**Figure 3:**
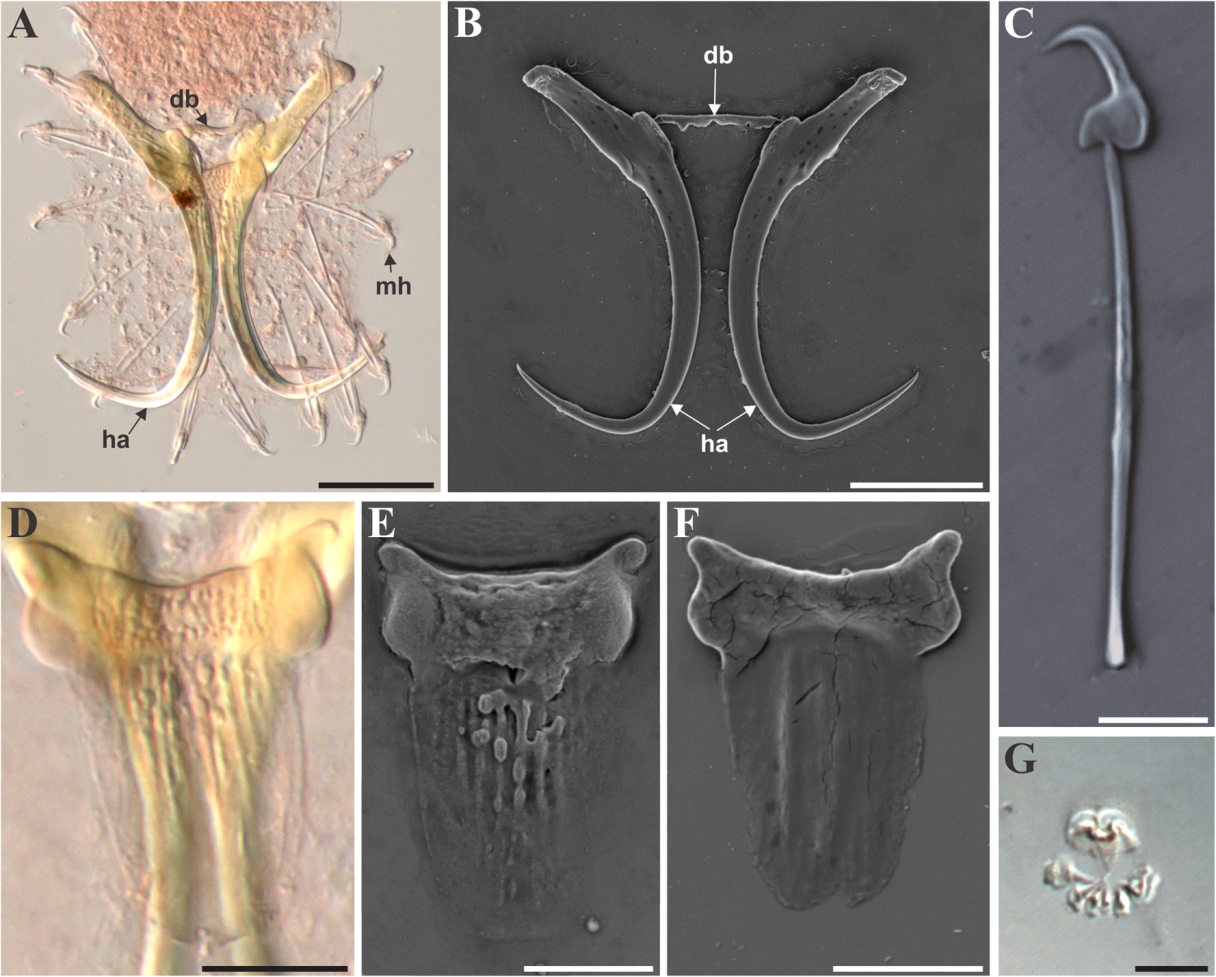
Light (LM) and scanning electron (SEM) micrographs of the haptoral sclerites and male copulatory organ (MCO) of *Gyrodactylus magadiensis* n. sp. (A) Haptoral sclerites with hamulus (ha), dorsal bar (db), and marginal hooks (mh), GAP (LM); (B) hamulus (ha) and dorsal bar (db) after soft tissue digestion (SEM); (C) isolated marginal hook (SEM); (D) dorsal bar (LM); (E) dorsal view of dorsal bar (SEM); (F) ventral view of dorsal bar (SEM); (G) male copulatory organ (MCO) with large central spine and six spinelets, two large and four small (LM). Scale bars – (A and B) 20 μm; (C and G) 5 μm; (D–F) 10 μm.

Description based on 24 individuals. Specimens 267.1 (202–387.2) long, and 65 (41.4–100.1) wide at level of anterior beginning of uterus. Pharyngeal bulb 21.1 (13.8–29.7) long, with anterior bulb 19.7 (13.6–28.2) wide and posterior bulb 20.7 (14.7–32.3) wide. Intestinal crura not spreading further than anterior edge of testes. Male copulatory organ (MCO) 11.4 (9.6–13.9) long and 8.5 (6.9–10.2) wide, situated posteriorly to pharyngeal bulb, armed with one central spine and six spinelets (two large and four small) (Figs. 2C, 3G).

Hamuli (Figs. 2A, 3A and B) slender, 62.8 (53.6–73.7) long; shaft even more slender 38.6 (34.8–43.6) long; narrow point 24.2 (19.9–29.5) long ending in a sharp point. Hamulus not sharply curved, aperture angle 59.9° (50.1°–78.1°); root straight, 22.3 (16.9–29.9) long. Dorsal bar simple, 13 (9.9–17.78) long and 2.6 (2.2–3.6) wide (Figs. 2A, 3A and B). Ventral bar 27.5 (22.6–33.3) long, 20.8 (14.6–26.4) wide; ventral bar processes small, anterolateral, rounded and slightly curved outward, 3 (2–4) long; ventral bar membrane ovoid, tongue-shaped with notch centrally at posterior, 18.7 (15.9–22.8) long (Figs. 2A, 3D–F). Marginal hooks 27.7 (23.4–30) long; hook shaft 23.1 (18.9–26) long with slight rounded swelling terminally (distally); marginal hook sickle slightly curved and tilted forward, 5.4 (4.7–6.1) long; point long and continuously curved from sickle, 4 (3–4.7) wide and distal width 3.4 (2.9–4) (Figs. 2B, 3C). Heel rounded strongly toward shaft; toe trapezoidal to square 1.8 (1.5–2.2) long, with sharp indentation on inferior edge between toe tip and shaft attachment point; long bridge prior to reaching marginal sickle shaft; long lateral edge of toe. Marginal hook aperture 4.4 (3.6–5.1); hook instep height 0.8 (0.62–1); filament loop 8 (6.5–9.2) long.

### Molecular identity of *Gyrodactylus magadiensis* n. sp.

**Figure 4:**
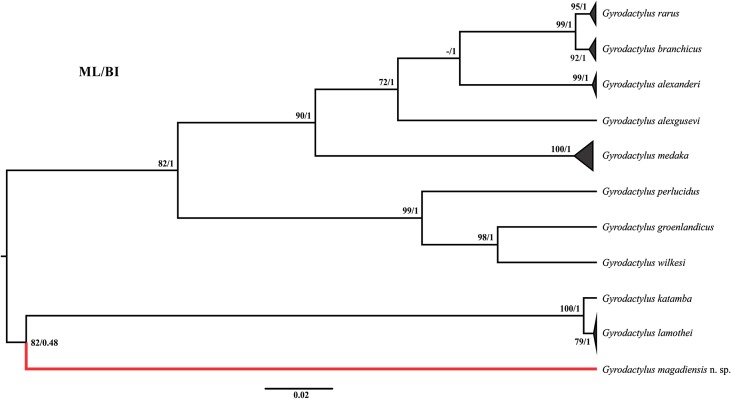
Evolutionary history of *Gyrodactylus magadiensis* n. sp. based on Bayesian Inference approaches using ITS sequences for selected gyrodactylids. Statistical support for Bayesian inference (BI) and maximum likelihood (ML) methods indicated at branch nodes with posterior probabilities and bootstrap support indicated, respectively (ML/BI).

All 10 specimens produced identical sequence data for the ITS fragment analysed. The amplified region was 882 bp in length, with the size of the 18S, ITS1, 5.8S, ITS2, and 28S rDNA fragments 24, 385, 158, 291, and 24 bp long, respectively. Alignment of the sequence to other data retrieved from GenBank produced a 1088 bp alignment, of which 636 positions were conserved, 446 variable, and 399 parsimony informative. *Gyrodactylus magadiensis* n. sp. was only distantly related to most other *Gyrodactylus* species (Table 4), most closely to *Gyrodactylus branchicus* Malmberg, 1964 (23.2%) and most distantly to *Gyrodactylus katamba* García-Vásquez, Guzmán-Valdivieso, Razo-Mendivil, and Rubio-Godoy 2018 (25.4%). Distances of 0.45–25.4% were observed between species included in these analyses, while intraspecific distances of 0.00–1.14% were seen. The latter would suggest that taxa with more than 1.14% sequence divergence are distinct species, indicating that sequences for distinct species with less than that (in this case *G. katamba* and *Gyrodactylus lamothei* Mendoza-Palmero, Sereno-Uribe and Salgado-Maldonado, 2009, and *G. branchicus* and *G. rarus* Wagener, 1910) need to be revised to produce a robust criteria to identify species based on ITS rDNA. Topologies of phylogenetic analyses based on ML and BI methods produced similar results, thus a single combined tree is shown in Figure 4. In all cases, *G. magadiensis* n. sp. formed a distinct, well supported linage from its congeners. *Gyrodactylus magadiensis* n. sp. appeared to be most closely related to a clade of *G. katamba* and *G. lamothei* in all cases.

**Table 4:** Sequence divergence (%) based on average uncorrected *p*-distance separating *Gyrodactylus magadiensis* n. sp. (values in bold) from other *Gyrodactylus* species. Intraspecific distances indicated by shaded cells.

Species	Accession		1	2	3	4	5	6	7	8	9	10	11	12	13	14	15	16	17	18	19	20	21
*G. magadiensis* n. sp.	MN738699	**1**	–																				
*G. branchicus*	AY061977	**2**	**23.20**	–																			
*G. branchicus*	AF156669	**3**	**23.44**	0.31	–																		
*G. rarus*	AY061976	**4**	**23.32**	0.73	1.05	–																	
*G. rarus*	FJ435193	**5**	**23.57**	0.73	1.05	0.21	–																
*G. rarus*	AY338445	**6**	**24.28**	0.75	1.07	0.21	0.00	–															
*G. perlucidus*	FJ435202	**7**	**23.55**	14.91	14.91	14.91	15.13	15.53	–														
*G. medaka*	LC368478	**8**	**23.54**	12.18	12.08	12.08	12.08	12.39	16.96	–													
*G. medaka*	LC368475	**9**	**23.66**	12.61	12.50	12.50	12.50	12.83	17.07	1.14	–												
*G. medaka*	LC368479	**10**	**23.78**	12.71	12.61	12.61	12.61	12.93	17.18	1.14	0.21	–											
*G. medaka*	LC368477	**11**	**23.78**	12.71	12.61	12.61	12.61	12.93	17.18	1.04	0.10	0.10	–										
*G. alexgusevi*	AY061979	**12**	**24.04**	9.64	9.96	9.64	9.64	9.89	17.63	14.16	14.38	14.48	14.48	–									
*G. groenlandicus*	KJ095104	**13**	**24.03**	15.77	15.88	15.88	15.88	16.25	8.67	17.26	17.26	17.26	17.36	18.58	–								
*G. alexanderi*	JN695633	**14**	**24.16**	7.25	7.35	7.14	7.14	7.33	16.20	12.93	13.25	13.14	13.14	10.98	17.54	–							
*G. alexanderi*	JF836144	**15**	**24.16**	7.14	7.25	7.04	7.04	7.22	16.09	13.04	13.35	13.25	13.25	10.88	17.36	0.10	–						
*G. alexanderi*	FJ435201	**16**	**24.27**	7.15	7.26	7.05	7.05	7.23	16.11	13.07	13.39	13.28	13.28	10.89	17.65	0.00	0.00	–					
*G. wilkesi*	LT719091	**17**	**24.97**	15.59	15.71	15.71	15.71	15.71	8.99	18.57	18.80	18.80	18.91	18.66	5.98	17.13	17.13	17.04	–				
*G. lamothei*	KX555668	**18**	**25.23**	22.98	23.09	22.86	23.09	23.09	22.70	23.22	22.99	22.99	22.99	22.44	23.76	22.06	22.17	22.08	23.22	–			
*G. lamothei*	KX555666	**19**	**25.30**	23.05	23.17	22.94	23.17	23.17	22.79	23.29	23.06	23.06	23.06	22.51	23.74	22.13	22.25	22.16	23.20	0.00	–		
*G. lamothei*	KX555667	**20**	**25.33**	23.08	23.19	22.96	23.19	23.19	22.82	23.32	23.09	23.09	23.09	22.54	23.77	22.16	22.27	22.18	23.23	0.00	0.00	–	
*G. katamba*	KR815854	**21**	**25.36**	23.00	23.11	22.88	23.11	23.14	22.97	23.12	22.89	22.89	22.89	22.46	24.03	22.08	22.20	22.11	23.29	0.45	0.56	0.56	–

### Differential diagnosis

In comparison to other *Gyrodactylus* species described from African cichlid fishes, the marginal hooks of *G. magadiensis* n. sp. are most similar to those of *Gyrodactylus cichlidarum* Paperna, 1968, *Gyrodactylus yacatli* García-Vásquez, Hansen, Christison, Bron and Shinn, 2011 and *Gyrodactylus ulinganisus* García-Vásquez, Hansen, Christison, Bron and Shinn, 2011 in that the sickle is smoothly curved (except *G. yacatli*) and the toe is almost square. The marginal hooks of the new species can be distinguished from these species in that the toe is more pronounced than in *G. cichlidarum* and *G. ulinganisus*; the indentation on inferior edge between the toe tip and shaft attachment point is more pronounced than in *G. cichlidarum* and *G. ulinganisus*; the bridge prior to reaching the marginal sickle shaft is longer than in *G. cichlidarum* and *G. ulinganisus*; the lateral edge of the toe is longer than in *G. yacatli*; and the sickle is angled forward (similar only to *G. yacatli*). The heel of *G. magadiensis* n. sp. is also notably rounded, only slightly similar to that of *Gyrodactylus thysi* Vanhove, Snoeks, Volckaert and Huyse, 2011, *Gyrodactylus thlapi* Christison, Shinn and van As, 2005 and the illustration of *Gyrodactylus niloticus* Cone, Arthur and Bondad-Reantaso, 1995 by Cone et al. [9] (junior synonym of *G. cichlidarum* [15]).

In terms of the ventral bar, *G. magadiensis* n. sp. can be differentiated from other *Gyrodactylus* species infecting African cichlids based on the distinct tongue shape of the membrane, medial notch in posterior of membrane, rounded lateral ends of the bar itself, and anterolateral processes rounded and slightly curved outward. These features are most similar to the ventral bar of *G. cichlidarum*, but specifically the shape of the membrane can easily distinguish these species. The long and narrow nature of the hamuli are reminiscent of those of *G. malalai*, *Gyrodactylus ergensi* Přikrylová, Matějusová, Musilová and Gelnar, 2009 and *Gyrodactylus nyanzae* Paperna, 1973. However, the hamuli of *G. magadiensis* n. sp. can be distinguished by the lack of an indentation of the root above the attachment of the dorsal bar as in *G. malalai* and *G. ergensi*, and the more robust root in comparison to *G. nyanzae*. The MCO of *G. magadiensis* n. sp. has six spinelets, whereas most of the other species for African cichlids have 4, 5 or 7 (with the exception of *Gyrodactylus shariffi* Cone, Arthur and Bondad-Reantaso, 1995 and *G. cichlidarum* which can have six).

## Discussion

*Gyrodactylus magadiensis* n. sp. is the second record of a *Gyrodactylus* species infecting a cichlid from Kenya. However, this is the first gyrodactylid to be described from the extreme conditions of Lake Magadi and from *Alcolapia grahami*. The ability of *G. magadiensis* n. sp. to persist and thrive in the extreme condition of Lake Magadi is truly impressive. Although *G. salinae* survives in the extreme salinity and temperature of Cervia Saline in Italy [48], this species is genetically very distant from *G. magadiensis* n. sp. and the morphologies of the species do not share many similarities. It is thus unlikely that these species, which are both able to survive extreme conditions, originated from the same lineage, indicating that this adaptation has occurred at least twice convergently.

Based on both morphology and genetic data, the material studied here is markedly distinct from all other information available for African gyrodactylid monogeneans. Regarding the genetic identity of *G. magadiensis* n. sp., a gyrodactylid infecting a marine Gasterosteiformes host (Gasterosteidae) collected in Finland and Belgium is the most closely related (23.2%) based on uncorrected *p*-distances, while none of the sequences for *Gyrodactylus* species from African cichlids were identified as close relatives using BLASTn, even though most of these African species have representative sequence data. In fact, it would appear that most of the species identified as close relatives to *G. magadiensis* n. sp. are marine species, which is puzzling. However, as can be seen from the topology of the phylogenetic analyses shown in Figure 4, *G. magadiensis* n. sp. groups with a clade of *G. lamothei* and *G. katamba*, both species from freshwater systems in Mexico. This association is only weakly supported by BI analyses (0.48), but more strongly by ML approaches (82%).

## Conclusion

*Gyrodactylus magadiensis* n. sp. is described here on the basis of its morphology and genetic identity. The species can be distinguished from congeners parasitising other African cichlids based on the comparatively long and narrow hamuli, a ventral bar with small rounded anterolateral processes and a tongue-shaped posterior membrane, and marginal hooks with slender sickles which are angled forward, a trapezoid to square toe, rounded heel, a long bridge prior to reaching the marginal sickle shaft, and a long lateral edge of the toe. Genetically, this species is distinct from all other monogenean species, with more than 23.2% pairwise divergence between it and its closest relatives.
